# Spatio-temporal assembly of functional mineral scaffolds within microbial biofilms

**DOI:** 10.1038/npjbiofilms.2015.31

**Published:** 2016-03-02

**Authors:** Yaara Oppenheimer-Shaanan, Odelia Sibony-Nevo, Zohar Bloom-Ackermann, Ronit Suissa, Nitai Steinberg, Elena Kartvelishvily, Vlad Brumfeld, Ilana Kolodkin-Gal

**Affiliations:** 1 Department of Molecular Genetics, Weizmann Institute of Science, Rehovot, Israel; 2 Department of Chemical Research Support, Chemical Research Support, Weizmann Institute of Science, Rehovot, Israel

## Abstract

Historically, multicellular bacterial communities, known as biofilms, have been thought to be held together solely by a self-produced extracellular matrix. Our study identified a novel mechanism maintaining *Bacillus subtilis* and *Mycobacterium smegmatis* biofilms—active production of calcite minerals. We studied, for the first time, the effects of mutants defective in biomineralization and calcite formation on biofilm development, resilience and morphology. We demonstrated that an intrinsic rise in carbon dioxide levels within the biofilm is a strong trigger for the initiation of calcite-dependent patterning. The calcite-dependent patterns provide resistance to environmental insults and increase the overall fitness of the microbial community. Our results suggest that it is highly feasible that the formation of mineral scaffolds plays a cardinal and conserved role in bacterial multicellularity.

## Introduction

Biofilms are multicellular communities that were so far thought to be held together solely by a self-produced organic extracellular matrix.^1–3^ *Bacillus subtilis* is a Gram-positive soil bacterium, well known for its ability to form robust and architecturally complex biofilms.^4^ *B. subtilis* biofilms are characterised by a high degree of wrinkling, which have been considered a direct read-out of organic matrix production. The matrix consists of exopolysaccharides, produced by enzymes encoded by the 15-gene *epsA-O* operon (henceforth *eps*), and amyloid-like fibres, specified by the *tapA-sipW-tasA* operon (henceforth *tapA*).^5–7^ In addition, the operon *ywqC-F* encodes for the tyrosine kinase PtkA,^8^ as well as for the production of acidic exopolysaccharides, yet its exact physiological role remains to be determined.^9^

Organic extracellular matrix production as a means of cell–cell and cell–substrate adhesion has been extensively studied.^2^ However, study of the possible role of mineral scaffolds in providing rigidity to bacterial biofilms has been neglected. Among the most common biogenic minerals formed are calcite and aragonite, which support the three-dimensional (3D) organisation of mollusks, echinoderms, calcisponges, corals, certain algae and others.^10,11^ Precipitation of calcium carbonate requires free Ca^2+^ as well as bicarbonate (HCO_3_^−^).^12^ Bicarbonate is a product of CO_2_ hydration (CO_2_+H_2_O↔HCO_3_^−^+H^+^), where the source of CO_2_ can be a byproduct of bacterial metabolism or of the immediate environment.^12–14^ Several independent groups demonstrated accumulation of calcium carbonate within bacterial biofilms.^12,15^ One fascinating example is calcite precipitation in *Proteus mirabilis*, *Proteus vulgaris* and *Providencia rettgeri* where crystal deposition in their biofilms blocks catheters.^15,16^ Importantly, the physiological role of mineral deposits within biofilms remains to be determined.

Here, we asked whether the control of calcium carbonate minerals can structurally support morphogenesis of bacterial colonies. We demonstrate that a mature 3D structure of mineral scaffolds holds the extracellular matrix and the bacterial cells together. Furthermore, we show that calcite-dependent morphogenesis is a conserved phenomenon, occurring in an additional genetically distant soil bacterium, *Mycobacterium smegmatis*.

## Results

### Mineral precipitation by *B. subtilis* results in complex colony morphology

**Figure 1:**
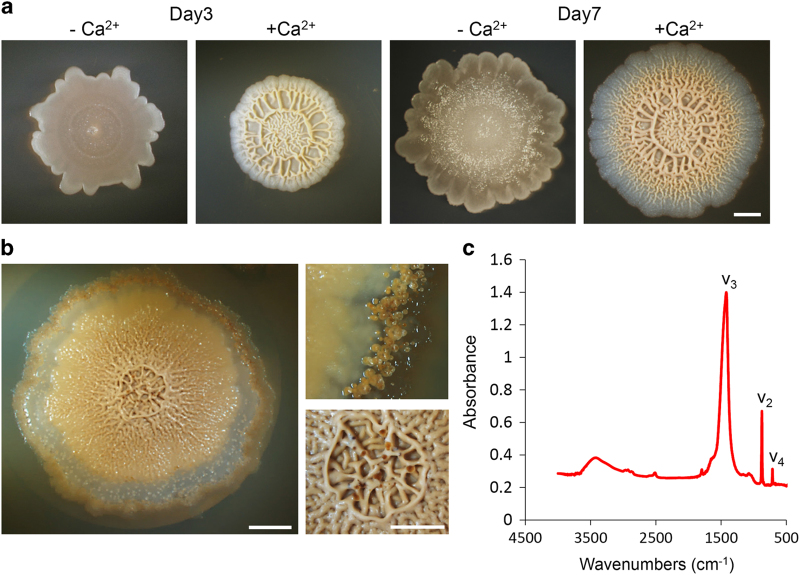
Complex colony morphology correlates with calcite precipitation in *Bacillus subtili*s. (**a**–**c**) Top view of a colony of an undomesticated strain of wild-type *B. subtilis* (NCIB 3610). The colonies were grown on solid biomineralization-promoting medium without (**a**) or with (**a** and **b**) a calcium source, for 3 and 7 (**a**), 21 (**b**) days, at 30 °C, in a CO_2_-enriched environment. (**b**) Top view of a colony (left) and a magnification of calcite crystals at the periphery (right upper) or centre (right lower) of the colony. Images were taken by Stereo microscope with an objective of ×0.5 (**a** and **b**, left) or ×1 (**b**, right) Scale bar corresponds to 2 mm (**a** and **b**, left) or 100 μm (**b**, right). (**c**) The FTIR spectra of calcium carbonate minerals precipitated at the edges of the colony. *ν*_2_ and *ν*_3_ indicate characteristic vibrations. The results are of a representative experiment out of five independent repeats.

**Figure 2:**
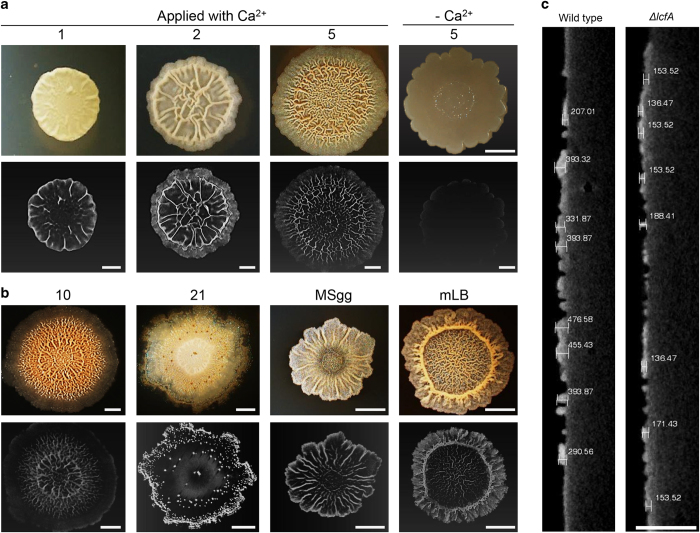
Calcite and amorphous calcium carbonate have a distinct spatio-temporal organisation within the biofilm. (**a**, **b**) Upper panel: Top view of a biofilm of wild-type *B. subtilis*. The biofilms were grown at 30 °C, in a CO_2_-enriched environment. (**a**) On solid biomineralization-promoting medium with a calcium source for 1, 2, 5 days or without a calcium source for 5 days. (**b**) The biofilms were grown on solid biomineralization-promoting medium with a calcium source for 10, 21 days or on MSgg and mLB medium for 5 days. Images were taken with a stereo microscope with an objective ×1. Scale bar corresponds to 500 μm. Lower panel: MicroCT images of *B. subtilis* biofilms. Scale bar corresponds to 2 mm. (**c**) Images representing the thickness of the calcium carbonate buildup underneath the wrinkles of wild-type and *lcfA* mutant. The images were obtained from the microCT by 2D slice cutting through the biofilm. Scale bar corresponds to 3.5 mm. The results are of a representative experiment out of three independent repeats.

To assess the possible roles of biomineralization in biofilm development, we grew wild-type *B. subtilis* cells on media in the presence or absence of calcium acetate as a calcium source. We observed flat, transparently white colonies, with no complex morphology in the absence of a calcium source (Figure 1a). Unexpectedly, in the presence of a calcium source, colonies were thicker and pigmented, with a complex morphology, and displayed a higher wrinkle count (Figure 1a), robustly evident starting from day 2. With time we observed formation of putative calcium carbonate crystals, located along the edges and within the colony (Figure 1b). Crystals appeared as granules (Figure 1b); no such granules were observed in the absence of calcium ions. Appearance of complex architecture and mineral granulations were also observed in the presence of an alternative calcium source, calcium chloride (Figure 2).

We further examined crystal formation under two environmental conditions: atmospheric air and a CO_2_-enriched environment (5–7% carbon dioxide at the expense of atmospheric oxygen). Calcium carbonate crystal formation was more robust in the CO_2_-enriched environment, when compared with that observed in atmospheric air. In contrast, under strict anaerobic conditions, where nitrate served as a terminal electron acceptor,^17^ calcite precipitation was eliminated, along with the ability of *B. subtilis* to produce a complex, wrinkled morphology (Supplementary Figure S1).

Next, Fourier transform infrared (FTIR) spectroscopy was used to identify the precipitated mineral observed within established biofilms. In this method, IR radiation is passed through a sample when some of it is absorbed by the sample and some of it is transmitted. The resulting spectrum, constitutes a unique fingerprint of the sample, represents the molecular absorption and transmission, and allows one to distinguish between different crystalline polymorphs of CaCO_3_: calcite, aragonite and vaterite. We removed the organic matter by a hypochlorite priming step, and analysis was performed on the inorganic matter only. The infrared calcite spectrum has three characteristic peaks in the region of 400–4000 cm^−1^, designated *v*_2_, *v*_3_ and *v*_4_. Calcium carbonate *ν*_3_ peak is expected at 1425 cm^−1^ for calcite, 1490 cm^−1^ for vaterite and 1475 cm^−1^ for aragonite. The calcium carbonate *ν*_2_ peak is expected at 875 cm^−1^ for calcite, 850 cm^−1^ for vaterite and 855 cm^−1^ for aragonite. Finally, the calcium carbonate* ν*_4_ peak is expected at 713 cm^−1^ for calcite, 750 cm^−1^ for vaterite and 715 cm^−1^ for aragonite.^18^ The FTIR spectra of the putative calcium carbonate minerals that were collected from the edges of the biofilm was typical of calcite, lacking a *ν*_1_ peak and containing a *ν*_2_ peak at 875 cm^−1^, *ν*_3_ peak at 1425 cm^−1^ and *ν*_4_ peak at 713 cm^−1^ (Figure 1c). The presence of calcite was confirmed in an independent X-ray diffraction analysis (Supplementary Figure S2).

### Calcium carbonate scaffolds are required for morphogenesis of biofilms

**Figure 4:**
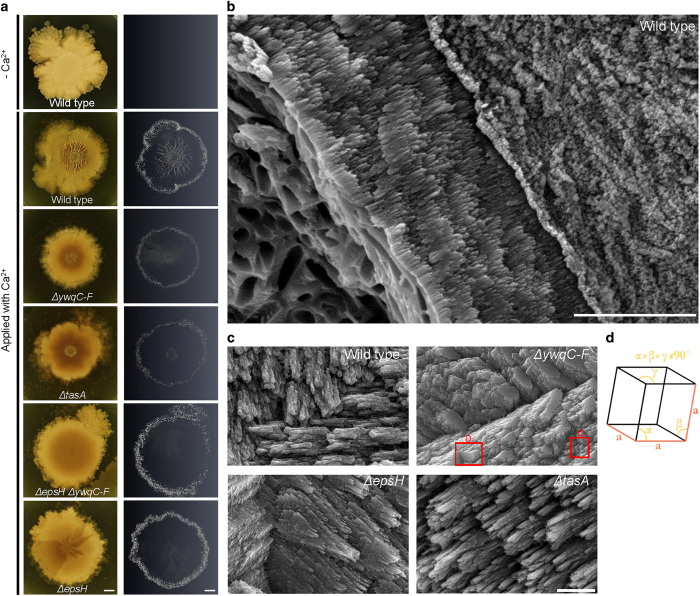
The extracellular matrix affects amorphous calcium carbonate (ACC) and calcite distribution. (**a**) Images represent biofilm phenotypes of a wild-type strain and its derivative biofilm formation mutants (mutants for extracellular matrix production): single mutants for *epsH, ywqC-F* and *tasA* and a double mutant for *epsH* and *ywqC-F*. Colonies were grown on solid biomineralization medium, in the absence or presence of a calcium source, for 19 days, at 30 °C. Left panel: images of biofilm, taken with a Nikon D3. Scale bar corresponds to 1 mm; Right panel: MicroCT images of the wild-type biofilm formation mutant strains. Scale bar corresponds to 2 mm. The results are of a representative experiment out of three independent repeats. (**b**) The surface morphology of a calcite mineral. An environmental scanning electron micrograph (ESEM) image of the calcite mineral extracted from the wild-type strain. The mineral was fractured, to expose its internal structure. Microscope magnification ×12,600; Scale bars correspond to 5 μm. (**c**) The acidic polysaccharides interact with the mineral phase. Environmental scanning electron micrograph (ESEM) images of calcite crystals extracted from the edges of the colonies of the wild-type strain and from its extracellular matrix mutant derivatives (*tasA*, *epsH* and *ywqC-F*). The crystals were extracted and washed to remove organic matter (see Materials and methods). Microscope magnification ×25,800. Scale bar corresponds to 2 μm. The results are of a representative experiment out of four independent repeats. The red square highlights a rhombohydral calcite crystal. (**d**) Model for spontaneous organisation of rhombohydral calcite crystal in solution.

The exact 3D distribution of calcite minerals within differentiated biofilms has not been analysed. We studied the distribution of minerals in the biofilm using micro-computed tomography (microCT). The microCT provides high-resolution assessments of density, geometry and microarchitecture of mineralised tissues and calcification.^19^ It uses X-rays to create cross-sections of a 3D-object that can later be used to recreate a virtual model. The microCT 3D images reinforced the observation that minerals were only synthesised in the presence of calcium ions in the media. Little or no mineral was produced by *B. subtilis* colonies grown on media lacking calcium ions (Figures 2a and 4a). In addition, the microCT images demonstrated that the mineral distribution was spatially and temporally distinct. The first area of biomineralization highly correlated with the biofilm wrinkles (Figures 2a and b). It appeared to occur in parallel to complex colony formation, and was discernible within 2 days. This area was composed mostly of amorphous calcium carbonate (ACC), as judged by X-ray diffraction. The thickness of the ACC at the centre of the biofilm was about 200 μm (Figure 2c). This accumulation could not be attributed to the reduced surface area or to the air-exposed surfaces of the wrinkles as the mineral accumulation was apparent both in the innermost and outermost layers of all wrinkles (Figure 2c). Thus it is not an incidental result of reduced dehydration and the concentration of mineral-forming ions in the centres of colonies. Namely, the mineral was situated within the wrinkles, and present in all layers of the biofilms. Moreover the calcium carbonate scaffolds below the wrinkles were observed in additional defined^3^ and rich biofilm media (Figure 2b). In all biofilm media, wrinkle morphology was associated with the presence of calcium. Thus, the formation of mineral scaffolds promotes complex morphology, regardless of the growth media composition. With time, calcite crystal emerged on top of the calcium carbonate scaffolds within the colony (Figure 1b). These results suggest that calcite accumulation may be enhanced by nucleation of ACC.

Over time, a spatially and temporally separate area of calcite crystal formation associated with the edges of the biofilm, appeared. This area was not visible in the first 5 days of culture (Figures 2a and b). Rather, it appeared a week later (Figures 1b and 2b), and was manifested by crystal-like particles of calcite, which formed a ring shape around the colony periphery, as well as on top of the thickest wrinkles. As calcite-dependent patterning occurred over long periods of time, we considered a possible role for spore formation in the calcification. However, a mutant defective in spore formation^20^ formed intact mineral patterns (Supplementary Figure S3). Thus, vegetative cells and their products are the dominant contributors to mineral accumulation. Intriguingly, 3 weeks post inoculation, the wrinkles as well as the calcium carbonate deposits, rapidly disassembled (Figure 2b).

Previous studies have suggested requirement of the *lcf* operon gene cluster (*lcfA-etfA,* designated hereby *lcf* operon) in biomineralization in the *B. subtilis* 168 domesticated strain, a biofilm-deficient strain, which is incapable of producing exopolysaccharides.^21,22^ Being a long-chain fatty acid ligase, *lcf*, together with other genes within the operon, were suggested to contribute to biologically controlled biomineralization by promoting membrane vesiculation.^21^ We therefore studied the effect of *lcf* operon deletion on biofilm development in our undomesticated strain. Remarkably, complex colony architecture associated with biomineralization only partially developed for the first 7 days, and later dramatically collapsed (Supplementary Figure S4). The *lcf* mutant colonies were significantly thinner (Figure 2c), suggesting that failed biomineralization leads to defects both in biofilm maturation and in 3D structuring. In addition to the significantly decreased mineral distribution within the defective wrinkles observed in the *lcf* operon mutant, calcite crystals formation was completely abolished (data not shown). The loss of calcite crystal edges led to increased spreading as a thin layer of the mutant, which covered the entire surface of the plate. Importantly, the *lcf* mutant had no effect on planktonic growth (Supplementary Figure S5).

### Intracellular control of the biomineralization-associated morphogenesis

**Figure 3:**
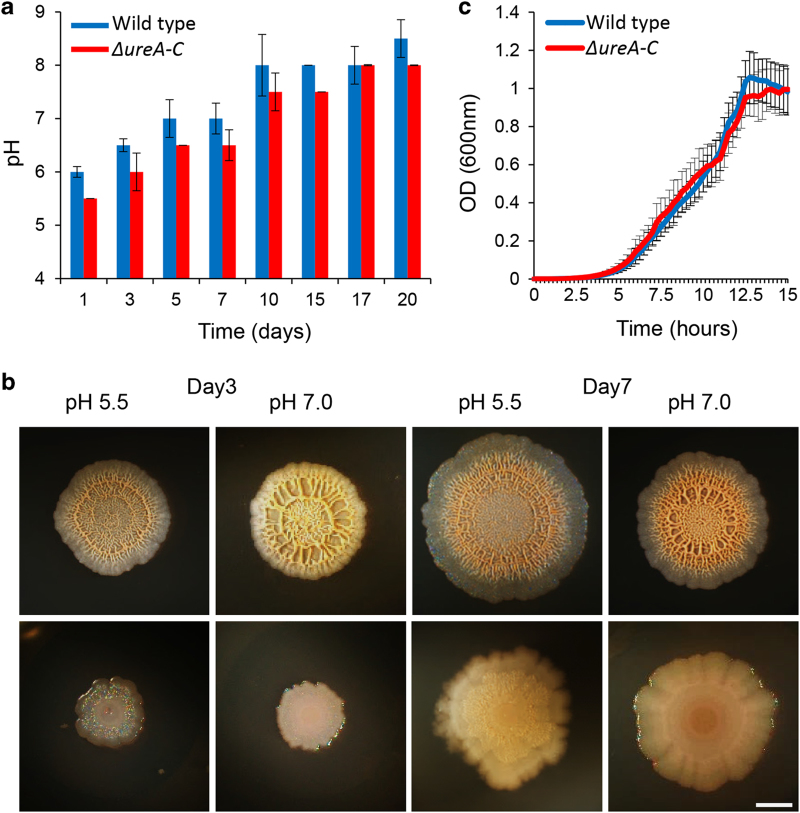
The biofilm cells promote the formation of a alkaline microenvironment, required for calcium carbonate precipitation and morphogenesis. (**a**, **b**) Wild-type and *ureA-C* mutant biofilms were grown on solid biomineralization-promoting medium with a calcium source, at 30 °C, in a CO_2_-enriched environment. (**a**) The intra-colony pH of wild-type (blue column) and *ureA-C* mutant strain (red column) on solid biomineralization-promoting medium, with acidic environment (pH 5.5). The results represent the averages and s.d. of three independent experiments. (**b**) Top view of a biofilm of wild-type *B. subtilis* (upper panel) and its *ureA-C* mutant (lower panel) derivative that grows in either an acidic (pH 5.5) or neutral (pH 7) environment, for 3 or 7 days. Images were taken by Stereo microscope with an objective ×0.5. Scale bar corresponds to 2 mm. The results are of a representative experiment out of three independent repeats. (**c**) Growth curves of wild-type (blue) and its *ureA-C* mutant derivatives (red) at 30 °C in liquid biomineralization-promoting medium. The results represent the averages and s.d. of six wells per strain, tested in three independent experiments.

Biomineralization has been thought to only be feasible at alkaline pHs. Alkaline environments promote generation of carbonate ions and negatively charge the functional groups on the bacterial surface.^12,23,24^ To test whether bacterial biofilms can buffer the microenvironment and enable efficient biomineralization, we examined the intra-colony pH as well as that of the surroundings of the colony, when grown in the absence or presence of a calcium source. The pH in each plate supplemented with a calcium source varied between acidic and alkaline environments (Figure 3). *B. subtilis* effectively precipitated calcium carbonate and formed wrinkles in acidic, neutral and alkaline conditions (Figure 3).

Surprisingly, under all growth conditions containing a calcium source, the intrinsic pH of the colony rose within 10 days to an alkaline pH (~8–8.5) (Figure 3a). Thus, controlled generation of a alkaline microenvironment facilitates calcium carbonate precipitation. In *B. subtilis,* urea is a nitrogenous compound that is generated by the degradation of arginine and purines.^25^ Hydrolysis of urea by urease is the most easily controlled mechanism of microbial calcium carbonate precipitation with potential to produce high quantities of carbonates within a short period of time: 1 mol of urea is intracellularly hydrolysed to eventually form bicarbonate, 1 mol of ammonium and hydroxide ions, which leads to increased pH. We hypothesised that a urease mutant, defective in controlling pH to promote biomineralization, would demonstrate fallings in colony morphogenesis. Thus, we tested a mutant in the *ure[A-C]* operon, encoding for urease production. Indeed, this mutant formed extremely featureless colonies, compared with the parental wild-type strain and demonstrated a poor ability to alkalise the neutral and acidic microenviornments within the biofilm (Figure 3). With time (from day 11), alkalisation of the environment enabling the formation of the crystals in the edge of the colony became evident in the urease mutant (Figure 3 and Supplementary Figure S6). Notably, this strain had no apparent defect in planktonic growth in biomineralization media (Figure 3c).

We then looked for additional genes that participate in calcite formation and morphogenesis in *Bacillus.* A BLAST search revealed three different putative homologues for carbonic anhydrase in the *B. subtilis* genome: *YvdA*, *YbcF* and *YtiB* (Supplementary Figure S7). Single, double or triple mutants of these genes had a very mild impact on calcite precipitation, presumably due to the alkaline microenvironment provided in biofilms, enabling spontaneous hydration of CO_2_ to bicarbonate. Notably, YvdA overexpression resulted in increased structure formation and pronounced calcite granules precipitation (Supplementary Figure S8). To summarize*, lcfA* operon and *ure[A-C]* encode intracellular enzymes, which may suggest an active role for the intracellular environment in mineral structuring.^26^ To further assess the importance of viable and active cells in mineral formation, we concentrated *B. subtilis* cultures, sterilised the culture to kill the cells without inducing lysis and plated them on biomineralization plates (Supplementary Figure S9). Indeed dead cells could not induce biomineralization.

In many cases, positively charged magnesium ions, in the presence of an excess of negatively charged surfaces, compete over the carbonate ions to promote formation of MgCO_3_ (magnesium carbonate) at the expense of CaCO_3_ (calcium carbonate). Thus, biologically induced biomineralization can be blocked on addition of magnesium chloride.^23^ It is notable that in the marine environment, microbial mats precipitate CaCO_3_ in the presence of access magnesium (up to fivefold higher than the concentration of calcium ions). However, this could be attributed to the solubility of magnesium ions compared with calcium ions, and is expected to be prevented by applying a less-soluble positively charged barium ions.^27^ When an excess of MgCl_2_ or BaCl_2_ was added to the growth media, both the accumulation of calcium carbonate within the wrinkles and wrinkle formation were not affected, demonstrating that mineralization within *B. subtilis* wrinkles is probably occurring in a buffered intracellular environment (Supplementary Figure S10). Furthermore, under all indicated cases a threshold accumulation of calcium carbonate preceded the formation of wrinkles (Supplementary Figure S8).

### Specific extrapolymeric substances interact with the mineral phase

It has been known that extracellular matrices absorb Ca^2+^ and promote calcium carbonate formation by providing additional nucleation sites.^14,28^ However, the exact extrapolymeric substance critical for biomineralization and structuring remains to be determined. Following our finding that a defect in biomineralization leads to a flaw in colony morphology, we wondered whether mineral absorption and assembly are related to the extracellular matrix substances secreted during biofilm formation.^2^ We examined mutants in the production of distinct extracellular matrix components, more specifically, the *tasA* mutant, impaired in production of secreted amyloids, *epsH* and *ywqC-F* mutants, impaired in different exopolysaccharide synthesis pathways and a double *epsH* and *ywqC-F* mutant. In all the mutants, complex morphology, particularly, wrinkles, was compromised (Figure 4a). *tasA and ywqC-F* had a mild defect in colony architecture development (Supplementary Figure S11 for day 5 and Figure 4a for day 20), while the single *eps* and double *eps ywqC* mutants had severe defects in morphogenesis. Calcite mineral precipitates were observed at the edges of the colonies of all mutants (Figure 4a). In contrast, we found that calcium carbonate assembly and localisation within the wrinkles were significantly compromised in the matrix mutants. None of the mutations had any effect on planktonic growth (Supplementary Figure S5).

Generation of ACC beneath the wrinkles was most severely decreased in the double *eps* and *ywqC-F* mutant (Figure 4a). The contribution of amyloid fibres to mineral precipitation at the colony centre was more modest, as observed in the *tasA* mutant (Figure 4a). However, in multiple experiments, the patterning of mineral accumulation within *tasA* mutant colonies was defective, when compared with the wild-type strain. Importantly, mineral deposits within the colony closely correlated with the 3D wrinkled extensions, previously described as solely dependent on the extracellular matrix.^5^ Thus, 3D structure formation is seemingly intimately related to biomineralization. It is highly feasible that the ability of the extracellular matrix to facilitate and direct mineral deposition plays a critical role in stabilisation of the complex architecture.

### Distinct extracellular matrix components direct the growth of calcium carbonate deposits

Growth of calcite crystals occurs in layers.^29^ Their relative growth rates in the various directions may affect crystal shape and its morphology, and may be affected by the biogenic (organic) environment,^30^ and with the organic polymeric substances.^31^ Since each extracellular matrix mutant has a specific effect on the production of biologically secreted macromolecules, the analysis of crystal morphology in the different matrix mutants can facilitate characterisation of the nature of interactions between the macromolecules and the mineral phase. To this end, crystals were collected, washed and analysed using environmental scanning electron microscopy (ESEM). In all backgrounds, crystals contained rod-shaped pores that corresponded to the size of the bacteria (Figure 4b). Examination of the crystals generated by the wild-type strain revealed rough faces, along with a smooth and flat crystal face, demonstrating the direct consequence of the spontaneous atomic organization (Figure 4c). The crystals formed in the wild-type strain displayed elongated prismatic morphology instead of the *rhombohedral* morphology, the most common form of calcite.^32^ Thus, the secreted organic matter interferes with crystal growth. Mutants for *tasA* amyloids and *eps* exopolysaccharides also formed crystals with an elongated morphology, indicating that the organic matter interferes with crystal growth.

For the wild-type strain, as well as *eps* and *tasA* mutants, the crystals were growing in the C axis, and the organic matter mainly interfered with growth of the crystals towards this axis. The levels of interference were evaluated according to the elongation pattern of the crystal, i.e., if the extensions are thinner, then the inhibition is higher. The highest inhibition was visualised in calcite produced by the *tasA* mutant, secreting only polysaccharides. Consequently, the interaction of the exopolysaccharides with the mineral phase inhibited crystals growth in the most robust manner.

The *ywqC-F* mutant, defective in secretion of acidic exopolysaccharides, produced calcite in strikingly clear *rhombohedral* morphology,^33^ (Figure 4c), as it has only one plane of symmetry through four vertices, and six smooth rhombic faces. Therefore, in this mutant, the interference of the organic matter with the crystal growth was minimal (Figure 4d illustrates spontaneous assembly of calcite crystals in solution, reminiscent of crystal assembly in *ywqC-F* mutant biofilms).

**Figure 5:**
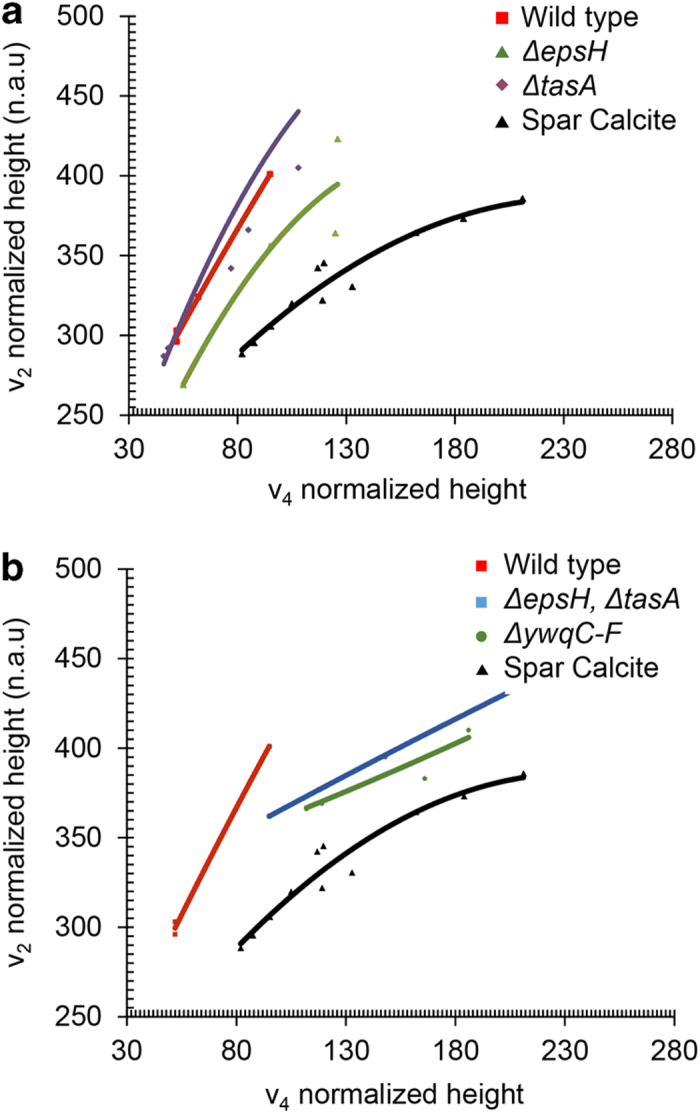
The degree of the local disorder of calcite is affected by the biofilm matrix. Plot of the *ν*_2_ versus *ν*_4_ peak heights of calcite crystals. The control sample is spar calcite. Each spectrum was normalised to the corresponding *v*_3_ peak height. For each type of calcite, data points correspond to successive grindings of the same specimen. The values along the trend line for the calcite crystal sample designate the width (FWHM) of the *v*_3_ peak. n.a.u., normalised absorbance units. (**a**) Calcite crystals purified from the wild type *epsH* and *tasA* mutant strains. Control sample is spar calcite (**b**) Calcite crystals purified from the wild type, a double mutant for *epsH*, *tasA* and *ywqC-F* mutant strains. Control sample is spar calcite.

To further assess the interaction between ECM components and mineral crystals, we used the FTIR to further investigate the nature of the crystals accumulated within *Bacillus* biofilms (Figure 5). Importantly, a comparison of width and height ratios of the *ν*_2_ and *ν*_4_ peaks allows to address in a more quantitative manner the differences in the level of crystallinity^34^ between the wild-type calcite crystals and the crystals formed in the mutants. The spectrum of crystalline calcite taken from the wild type had a clear and conserved ratio between the *ν*_2_ peak at 875 cm^−1^ and the *ν*_4_ peak at 750 cm^−1^. Importantly, the results lay on a control trend line produced by a geogenic single crystal of Spar calcite,^35^ which is known to have high atomic order over macroscopic length scales.^36^ The steepest slopes correspond to the wild type and the ECM mutants. Strikingly, a significant separation between the trend lines is evident. Thus, minerals produced by ECM mutants in a mutant impairing the *eps* operon (neutral polysaccharides), in a mutant for *tasA* (amyloid fibres) (Figure 5a), or in a mutant for both ECM components (Figure 5b), are altered in the molecular organisation of the calcite. In addition and in agreement with the morphology of the crystal observed by ESEM (Figure 4c), the trend line for the *ywqC-F* mutant is the closest to that of the single spare calcite crystal, grown *in vitro* (Figure 5). These measurements indicate that the levels of crystallinity and the level of the disorder within the crystals, is strongly affected by interactions between the non-organic mineral phase and the ECM.

### Genetic regulation of the extracellular matrix in biomineralization media

To assess the status of transcriptional regulation of biofilm formation during biomineralization, we monitored the expression levels of *sinI* using P_*sinI*_-*luciferase*. SinI is an antirepressor, antagonising the repressor SinR, and thereby regulates biofilm formation and extracellular matrix production.^37^ It is synthesised under the direct control of the master-regulator Spo0A.^38^ We tested the expression pattern in two different media; standard biofilm medium that was used previously to characterise P_*sinI*_-*luciferase* expression and biomineralization-promoting medium. *sinI* expression was extremely high in biomineralization media, even compared with the routinely used biofilm media (Supplementary Figure S12A). Thus, we asked which signalling pathway is responsible for *sinI* expression in biomineralization media. *sinI* expression was almost abolished in a mutant lacking *spo0A* (Supplementary Figure S12B). Spo0A is activated by phosphorylation via a phosphorelay involving five histidine kinases: KinA, KinB, KinC, KinD and KinE.^39^ We studied calcium-induced P_*sinI*_-*luciferase* expression in *kinA* and *kinB* genes pair, *kinC* and *kinD* gene pair double mutants and the *kinA kinB kinE* triple mutant (Supplementary Figure S12B). The calcium-induced expression in the *kinC, kinD* double mutant was delayed when compared with the wild type, but reached a comparable level of intensity within 24 h. *sinI* expression was most significantly reduced in the *kinA, kinB* gene pair mutant, suggesting that these redundant kinases^39^ are the main signal transducing kinases during biomineralization.

### Biomineralization media promotes resilience of bacterial biofilms from environmental insults

The formation of calcite reduces diffusion by 7 orders of magnitudes.^40^ Thus we asked whether mineral accumulation may limit penetration of antibacterial agents into bacterial biofilms. Recently, it was demonstrated that the formation of structure in *B.* *subtilis* is a barrier towards ethanol penetration into the colony.^41^ Thus, we compared the response of *Bacillus* biofilms treated with a frequently used antibacterial agent ethanol in conditions that either promote or inhibit biomineralization. Quite strikingly, mineral accumulation enhanced the resilience of bacterial biofilms to ethanol by 42-fold. Only 0.7% of the cells survived ethanol treatment in conditions not permitting mineral accumulation. When biomineralization was favourable, more than 30% of the biofilm cells resisted ethanol treatment (Supplementary Figure S13).

### Colony morphogenesis in *M. smegmatis* requires the formation of mineral scaffolds

**Figure 6:**
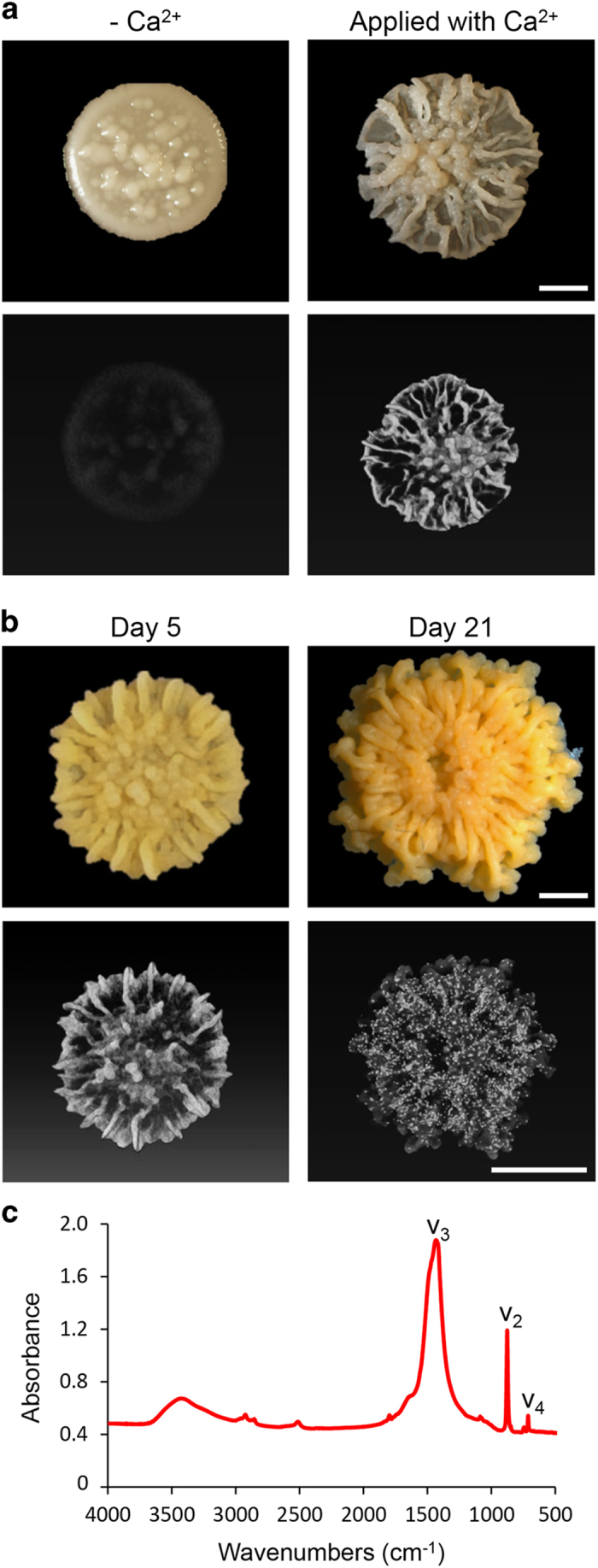
Biomineralization is required for formation of complex morphology in *Mycobacterium smegmatis*. (**a**, **b**) Upper panel: top view of the biofilm of the undomesticated *M. smegmatis*. The biofilms were grown on solid biomineralization-promoting medium without (**a**) or with (**a**, **b**) a calcium source, for 3 (**a**), 5, 21 (**b**) days at 30 °C, in a CO_2_-enriched environment. Images were taken with a Stereo microscope with an objective ×1. Scale bar corresponds to 500 μm. Lower panel: MicroCT images of the biofilms formed by *M. smegmatis*. Scale bar corresponds to 2 mm. The results are of a representative experiment out of three independent repeats. (**c**) The FTIR spectra of calcium carbonate minerals precipitated within the colony. The designation of the peaks as *v*_2_, *v*_3_ and *v*_4_ is marked. The results are of a representative experiment out of five independent repeats.

Following our observation that calcium carbonate scaffolds play a critical role in the development of *Bacillus* biofilms, we asked how general the role of biomineralization is in determining colony morphology in biofilm-producing bacteria. *M. smegmatis* is a Gram-positive soil bacterium capable of forming complex biofilms. It is genetically distinct from *B. subtilis* but closely related to highly pathogenic *Mycobacteria* species, like *M. leprea, M. tuberculosis and M. bovis*.^42^ *M. smegmatis* forms extremely complex morphologies over solid media.^43^ We hypothesised that complex colony morphology in *Mycobacterium* may dependent on biomineralization. We observed that, similarly to *B. subtilis, M. smegmatis* formed flat, transparent, white colonies, with no complex morphology in the absence of a calcium source (small colony with few wrinkles at the centre) over a 20-day inoculation period. In contrast, in the presence of a calcium source, colonies were dramatically thicker, with highly complex morphology, containing many wrinkles (Figure 6). The microCT analysis revealed a highly patterned biomineralization process that occurred only during pattern formation, and again, correlated with the start of biofilm wrinkles formation on day 3 (Figure 6a). The mineral was situated within the wrinkles and was present in all layers of the *M. smegmatis* biofilms. With time, calcium carbonate deposits within the wrinkles developed into numerous crystals (Figure 6b). The FTIR spectra of the putative crystals that were collected from the biofilm was typical of calcite, containing a *ν*_2_ peak at 875 cm^−1^, *ν*_3_ peak at 1425 cm^−1^ and *ν*_4_ peak at 713 cm^−1^ (Figure 6c).

## Discussion

James Shapiro proposed multicellularity nearly 20 years ago as a general bacterial trait. Shapiro's proposal in 1988 was based largely on observing colony morphogenesis.^44^ Bacterial morphogenesis is now considered an adaptive strategy for nearly all bacteria, among them *Actinobacteria, Myxococcus* and *Firmicutes*.^45^

Our study of *B. subtilis* identified an unexpected component critical to the maturation and maintenance of bacterial multicellular communities. We found that the formation of extracellular minerals and crystals plays a cardinal role in the patterning and morphogenesis of biofilms. Calcite minerals may serve as a load-bearing foundation in complex structures, promoting the stability of the overall structure. The incorporation of mineral component into the biofilm is expected to increase rigidity significantly, and thus increase the resistance to embedded cells to shear stresses and dispersal.

The active role of biofilm cells in promoting biomineralization was manifested in our finding that the proteins required for efficient biomineralization and morphogenesis of the bacterial biofilms were intracellular (Figures 2 and 3 and Supplementary Figure S4). An especially important determinant for mineral formation and structuring was the ability of urease-dependent metabolism in buffering both the intracellular environment and the extracellular environment, and to stimulate calcium carbonate accumulation (Figure 3). Applying an excess of magnesium or barium to compete over nucleation sites did not interfere with either morphogenesis or calcium carbonate accumulation (Supplementary Figure S10). Thus, we hypothesize that the initial events necessary for biomineralization are intracellular. The exact mechanism for the transport of calcite remains to be determined, and could resemble the formation of magnetite in the single cell level. Magnetite formation is characterised by ferromagnetic single-domain nanoparticles packaged in discrete vesicle compartments called magnetosomes, aligned by a bundle of filaments along the cell axis of the magnetotactic bacteria.^46–48^ While it is highly feasible that the mineral growth initiates within the cells or strongly requires a buffered intracellular environment, eventually the mineral is assembled to its final structure via complex interactions with the extracellular organic manner, particularly with the amyloid fibres (Figure 4a). Growth is primarily antagonised by negatively charged polysaccharides (Figure 4c). A model summarizing the roles of cellular and matrix contributions to mineral structuring is featured as Supplementary Figure S14.

Numerous studies of *B. subtilis* and additional biofilm formers established that complex colony architecture is dependent on the organic extracellular matrix,^2,4,6,49^ and thus hypothesised that the presence of the organic ECM components is sufficient for bacterial morphogenesis. We found that three elements are all necessary for robust bacterial development: functional mineral deposits, organic extracellular matrix mineral complexes and a cellular microenvironment promoting controlled pH. As a result, our understanding of the molecular mechanisms underlying colony morphogenesis has grown, and several new targets for biofilm inhibition were introduced. Our observations also agree with and shed light on the common findings that in many clinical and ecological settings biofilms precipitate calcium carbonate.^15,50^

Cells in biofilms are typically more resistant to antibiotics when compared with free-living bacteria.^51,52^ While this phenomenon is multifactorial, the ability of the matrix to reduce antibiotic penetration was often considered as an appealing explanation.^53^ Most of the antibiotics are water soluble. However, water diffusion coefficient changes dramatically in a mineral compared with a polysaccharide film. For example, water diffusion in polysaccharides film (cellulose) is 4–14×10^−8^ cm^2^/s, compared with the 2 order of magnitudes slower water diffusion in calcium carbonate—20×10^−6^ cm^2^/s.^54^ Furthermore, diffusion can be reduced by 7 orders of magnitudes in crystalline calcite.^40^ Thus, our finding that bacterial biofilms rely not only on organic material (extracellular matrix) but also on inorganic calcite mineral scaffolds has dramatic implications for possible therapies for biofilm-associated infections. A preliminary evidence for this hypothesis is manifested by our finding that *B. subtilis* biofilms are up to 42-fold more resistant to ethanol in conditions promoting biomineralization (Supplementary Figure S13).

Oxygen limitation was previously shown to have a pivotal role in wrinkles formation by several bacteria.^17,55,56^ We have shown that endogenous accumulation of carbon dioxide and calcium availability serve as an additional signal for extracellular matrix production. It is important to note that carbon dioxide accumulation from aerobic respiration occurs in the same conditions where diffusion rate and oxygen availability within the biofilms become limiting.^57^ Therefore, it is feasible that both phenomena exert synergistic pressure for bacterial morphogenesis. The formation of wrinkles composed of mineral scaffolds can serve two distinct complementary roles: to increase the availability of oxygen and to precipitate toxic carbon dioxide.

In conclusion/overall/ these results provide an unprecedented demonstration of orchestrated production of extracellular calcite scaffolds, which support morphogenesis in simple prokaryotes. Mineral scaffolds formation has a profound effect on the structural organisation of colony biofilms in both *B. subtilis* and *M. smegmatis*, demonstrating that biomineralization plays important conserved roles in the development of microbial biofilms.

## Materials and methods

### Strains

*B. subtilis* strains were derivatives of the wild-type strain NCIB 3610 that form robust biofilms ^3^ (Supplementary Table S1). Deletions were generated by long-flanking PCR mutagenesis. Transformation of *B. subtilis* PY79 with double-stranded PCR fragments, was done as described previously.^58^ The primer list is provided in Supplementary Table S2. The genomic DNA of PY79 was then transformed to NCIB 3610, resulting in the resistant strains.

The *M. smegmatis* MC^2^155 strain was kindly provided by Dr. Eyal Gur (Ben-Gurion University).^59^

### Strains, media and imaging

*B. subtilis* and *M. smegmatis* cells were grown on biomineralization-promoting solid medium—B4 medium (0.4% yeast extract, 0.5% glucose, 0.25% calcium acetate and 1.5% agar^21,60^) at 30 °C. As a control for the mineralization process, bacteria cells were grown on B4 plates without calcium acetate. *B. subtilis* cells were grown under two different environmental conditions: in an open box exposed to the atmospheric air conditions or in an enriched CO_2_ environment achieved by using the candle jar method.^61^ To achieve anaerobic conditions, *B. subtilis* was grown on B4 plates supplemented with nitrate (20 mM KNO_3_) as a terminal electron acceptor. The plates were incubated at 30 °C in a sealed box (BD GasPak EZ Anaerobe Pouch System, Becton, Sparks, MD, USA), for up to 21 days.

In addition, *B. subtilis* cells were grown on modified LB (mLB) medium^62^ where manganese(II) sulfate was replaced with manganese chloride and on MSgg solid medium^39^ (5 mM potassium phosphate, 100 mM morpholinepropanesulfonic acid, pH 7 [MOPS], 2 mM MgCl_2_, 50 μM MnCl_2_, 50 μM FeCl_3_, 700 μM CaCl_2_, 1 μM ZnCl_2_, 2 μM thiamine, 0.5% glycerol, 0.5% glutamate and 50 μg/ml (each) threonine, tryptophan and phenylalanine). Solid medium contained 1.5% Bacto agar. The plates were incubated at 30 °C in a CO_2_-enriched environment, up to 21 days. Solid medium (Luria–Bertani broth (LB) agar), used for growing bacterial strains for transformation, was applied with erythromycin (1 μg ml^−1^), kanamycin (5 μg ml^−1^, spectinomycin (50 μg ml^−1^) and tetracycline (10 μg ml^−1^) were used for selection in *B. subtilis*, as required. Ampicillin (100 μg ml^−1^) was used for selection in *Escherichia coli*, as required.

The colonies were observed using a Nikon D3 camera or a Stereo Discovery V20" microscope (Tochigi, Japan) with objectives Plan Apo S ×0.5 FWD 134 mm or Apo S ×1.0 FWD 60 mm (Zeiss, Goettingen, Germany) attached to a high-resolution microscopy Axiocam camera, as required. Data were captured using Axiovision suite software (Zeiss).

### Monitoring pH changes during biofilm development

pH changes of the *B. subtilis* biofilm were monitored using 10 μl of a universal indicator solution (Sigma, St Louis, MO, USA) outside and inside the colony, at different time points (days). The pH in each plate varied between acidic and alkaline environments. The pH in each plate was maintained by 1 M HCL and 1 M NaOH solutions.

### Calcium carbonate crystal production

To produce crystals, strains were streaked on biomineralization-promoting solid medium and incubated at 30 °C for up to 30 days. Crystals were visible after 15 days of incubation. Crystals were collected as described by Mahamid *et al.,*^63^ with some modifications: agar samples were slightly bleached with 6% sodium hypochlorite for 1 min to remove organic matter, washed with Milli-Q water (Merck KGaA, Darmstadt, Germany) twice and dehydrated in acetone.

### FTIR spectrophotometer analysis

FTIR spectra of the produced crystals were acquired in KBr pellets by using a NICOLET iS5 spectrometer (Thermo Scientific, Pittsburgh, PA, USA). A few milligrams of sample were homogenised and powdered in an agate mortar and pestle. About 0.3 mg were left in the mortar and mixed with about 40 mg of KBr and pressed into a 7 mm pellet using a manual hydraulic press (Specac, Orpington, UK ). Each sample was measured repeatedly, either by repetitive grinding of the same KBr pellet. Typically, a few seconds of regrinding were applied. Infrared spectra were obtained at 4 cm^−1^ resolution for 32 scans using a Nicolet 380 instrument (Thermo, Instruments LLC, Madison, WI, USA). The baselines for the height measurements of the *v*_2_, *v*_3_ and *v*_4_ peaks were determined as done previously.^64^ The *v*_2_, *v*_3_ and *v*_4_ heights were normalised to a *v*_3_ height of 1,000, corresponding to 1.0 a.u.^65^

The X-ray diffraction was performed as done previously.^66^

### MicroCT and X-ray images

To image the distribution of calcite minerals within the colony, colonies on a small petri dish (60×15 mm) were placed in a microCT (MICRO XCT-400, Zeiss X-ray microscopy, Pleasanton, CA, USA). Tomography was carried out using a micro-focused source set at 20 kV and 2 mA; 750 images were taken with a 5-s exposure time. The final voxel size was 31 μm. Raw data were reconstructed with Zeiss software (Zeiss) that uses a filtered back-projection algorithm. 3D volume rendering (maximum intensity projection) was carried out with Avizo software (VSG, Hillsboro, OR, USA).

### Electron microscopy analysis

The dry crystals produced from the biofilm (see ‘calcium carbonate crystal production’) were mounted onto aluminium stubs using double-sided carbon tape. The crystals were fractured to allow imaging of the fracture surface. The crystals were sputter coated with gold-palladium (Edwards, S150, Wilmington, MA, USA) and visualised using a secondary electron detector in an ESEM (XL30 ESEM, PHILIPS, Eindoven, Netherlands).

### Real-time measurements of growth and gene expression using luciferase

Cultures were grown in LB medium overnight. The cultures were diluted 1:100 into one of three medium conditions: standard biofilm medium (MSgg), biomineralization-promoting solid medium, with or without calcium acetate (B4), as required. About 150 μl of each culture were plated onto a 96-well polystyrene visiplate (Thermo). The cultures were grown for 15–30 h at 30 °C in a plate reader (BioTek, Winooski, VT, USA), and the optical density at 600 nm (OD_600_) and luminescence (sensitivity setting, 200) were measured every 20 min. The data shown in the figures are averages for five repeat wells from a single representative experiment out of three, where luminescence values were normalised to the optical density (OD_600_).
