# Supplementary material for: Spatio-temporal assembly of functional mineral scaffolds within microbial biofilms
Source: NPJ Biofilms Microbiomes. 2016 Mar 2;2:15031–. doi: 10.1038/npjbiofilms.2015.31 (PMC5515261; doi:10.1038/npjbiofilms.2015.31)
Supplement: Supplementary Information [file npjbiofilms201531-s1.pdf]

Spatio-temporal Assembly of Functional Mineral Scaffolds within Microbial Biofilms

Yaara Oppenheimer-Shaanan, Odelia Sibony-Nevo, Zohar Bloom-Ackermann, Ronit Suissa, Nitai Steinberg, Vlad Brumfeld, Elena Kartvelishvily, and Ilana Kolodkin-Gal*

*Corresponding author

Supplementary information

Supplementary information content:

Supplementary figures (S1-S14)

Supplementary Figure Legends (S1-S14)

Supplementary table (S1-S2)

Supplementary references

Supplementary Figure S1

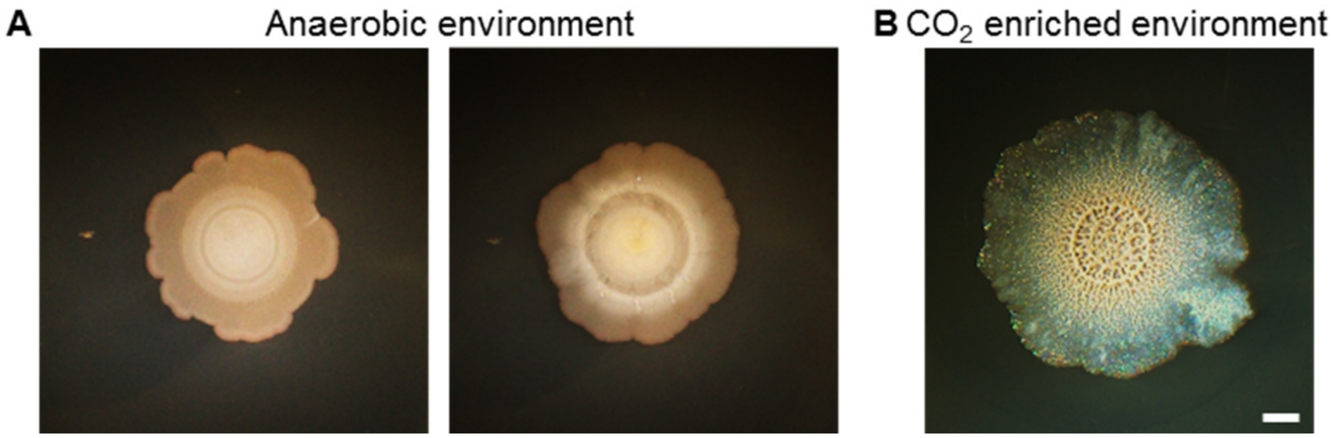

Supplementary Figure S2

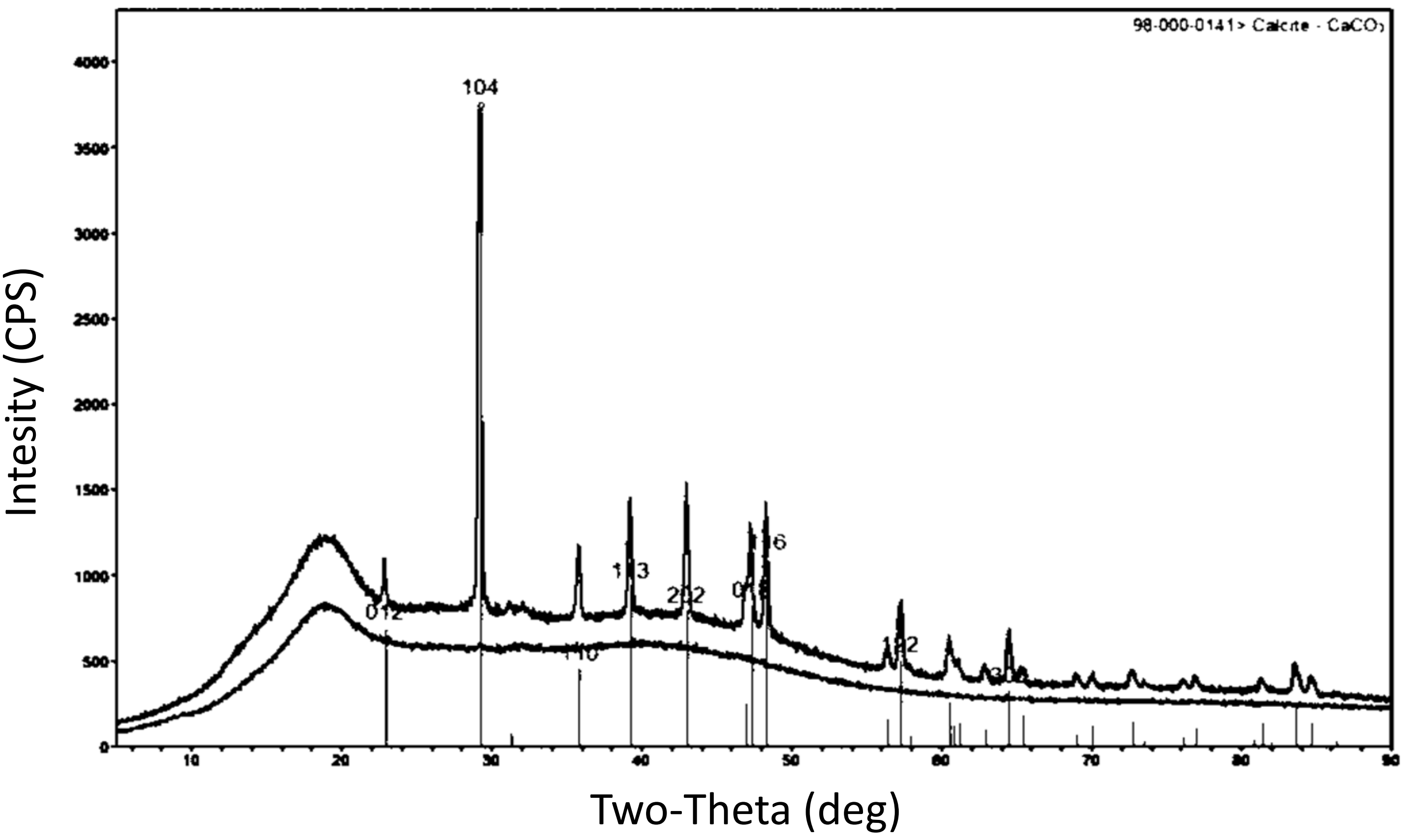

Supplementary Figure S3

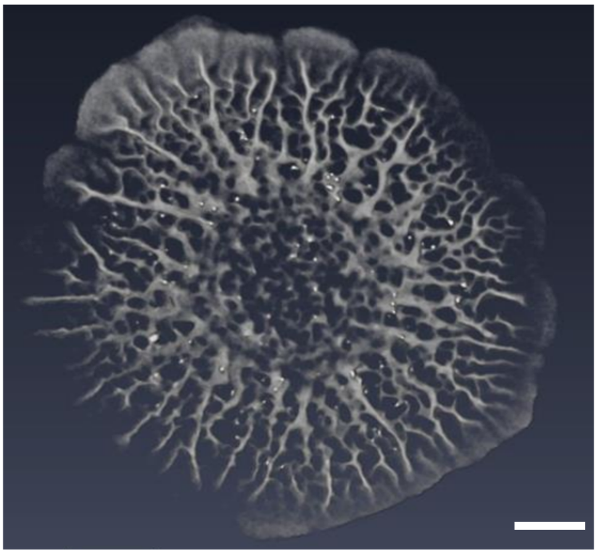

Supplementary Figure S4

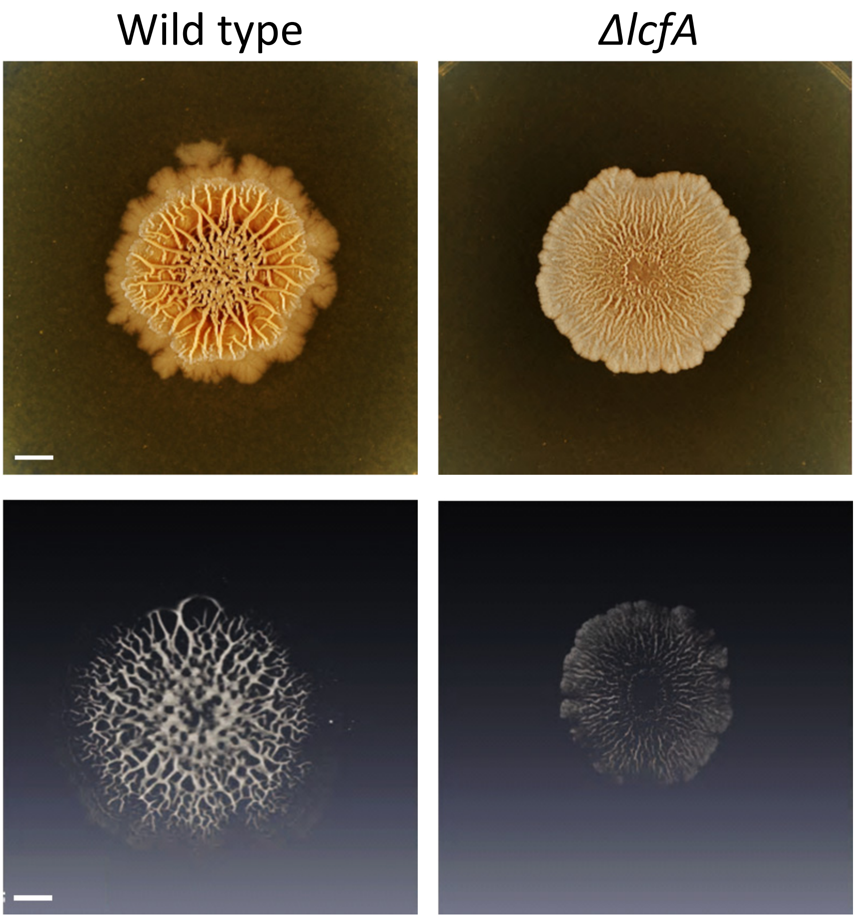

Supplementary Figure S5

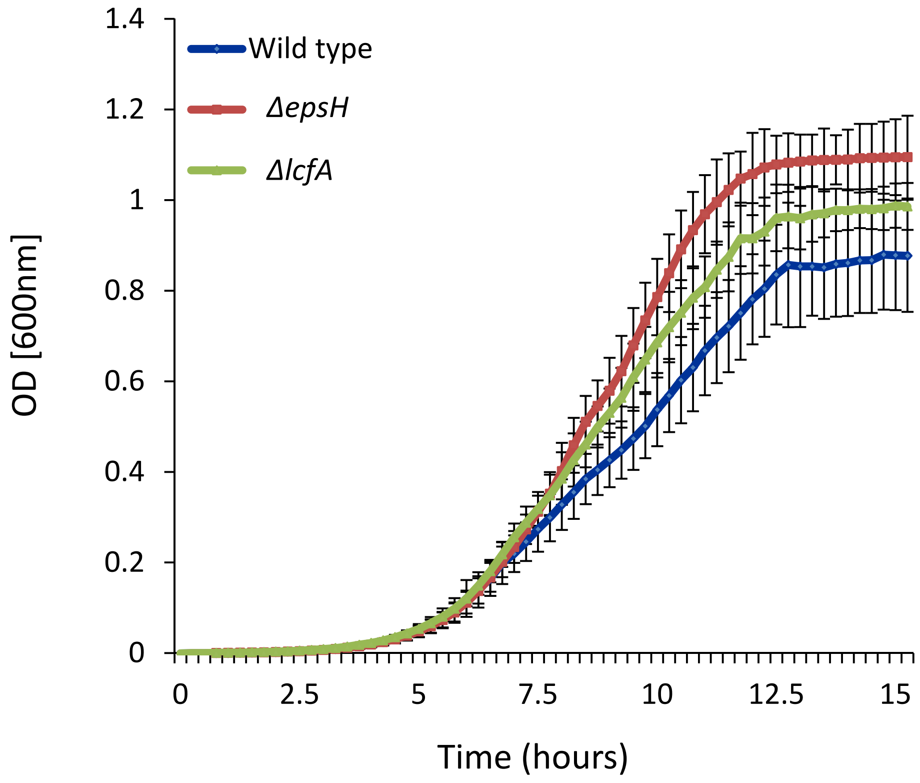

Supplementary Figure S6

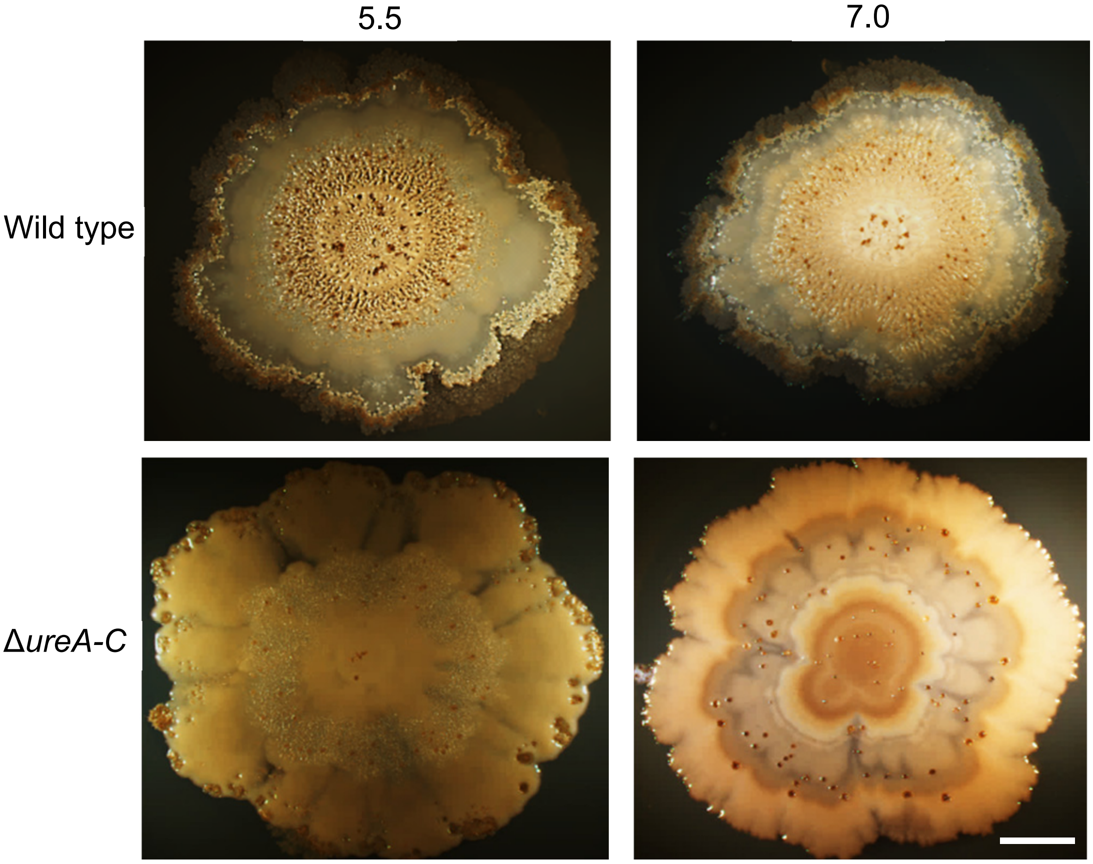

Supplementary Figure S7

ytiB

```
B. megaterium  1    msllqdvlefnkkfveekkyelyetskfpdkkmvilscmdtrlvellphalnlrngdvki  60
                    msll d+lefnk f e+++ye y+tskfpdkkm ilscmdtrlvellpha+nlrngdvki
B. subtilis    1    msllndilefnktfteqreyekyqtskfpdkkmailscmdtrlvellphamnlrngdvki  60

B. megaterium  61   vknagalvshpfgsimrsilvavyelqadevcvighhdcgagklqaepflekvrakgisd  120
                    +k+agalv+hpfgsimrsilvavyel adevcvighhdcg  k+ ++  lek++a+gi +
B. subtilis    61   iksagalvthpfgsimrsilvavyelnadevcvighhdcgmskissksmlekikargipe  120

B. megaterium  121  evintieys-mdlkqwltgfdsveetvqhsvetirnhplfskdtpvhglvidpntgkldv  179
                    e i ti+ys +d  qw   fdsve +v+ sv+ i++hplf ++ pvhglvidp tgkld+
B. subtilis    121  erietikysgvdfdqwfksfdsveasvkdsvdvikhhplfpenvpvhglvidpktgkldl  180

B. megaterium  180  vvngy  184
                    +vngy
B. subtilis    181  ivngy  185
```

ydvA

```
B. megaterium  1     msllqdvlefnkkfveekkyelyetskfpdkkmvilscmdtrlvellphalnlrngdvki  60
                     m   l  +le n++fv ekkye y+t+kfp kk+vi++cmdtrl ellp a+ l+ngd ki
B. subtilis    4     mvsltsilehnqrfvsekkyepykttkfpskklvivtcmdtrltellpqamglkngdaki  63

B. megaterium  61   vknagalvshpfgsimrsilvavyelqadevcvighhdcgagklqaepflekvrakgisd  120
                    vknaga+vshpfgs+mrsilva+yelqa+evc++ghh+cg   l a   lek + +g+ d
B. subtilis    64   vknagaivshpfgsvmrsilvaiyelqaeevcivghhecgmsglnassilekakergved  123

B. megaterium  121  evintieys-mdlkqwltgfdsveetvqhsvetirnhplfskdtpvhglvidpntgkldv  179
                      +n +  + +dlk wltgf svee+v hsv  i+nhpl  k  pvhglvi p tgkldv
B. subtilis    124  sclnlltsagldlktwltgfhsveesvshsvnmiknhpllpkkvpvhglvihpetgkldv  183

B. megaterium  180  vvngyea  186
                    v+ngye
B. subtilis    184  vingyet  190
```

Supplementary Figure S8

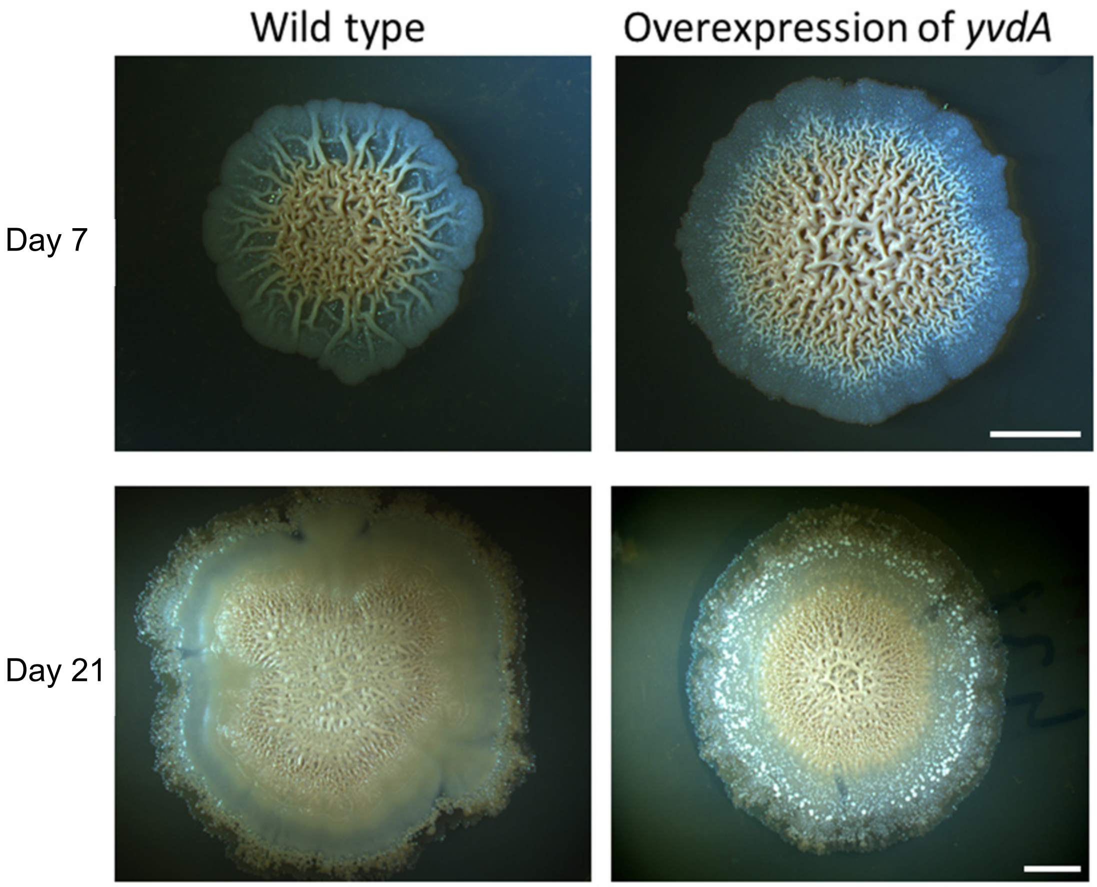

Supplementary Figure S9

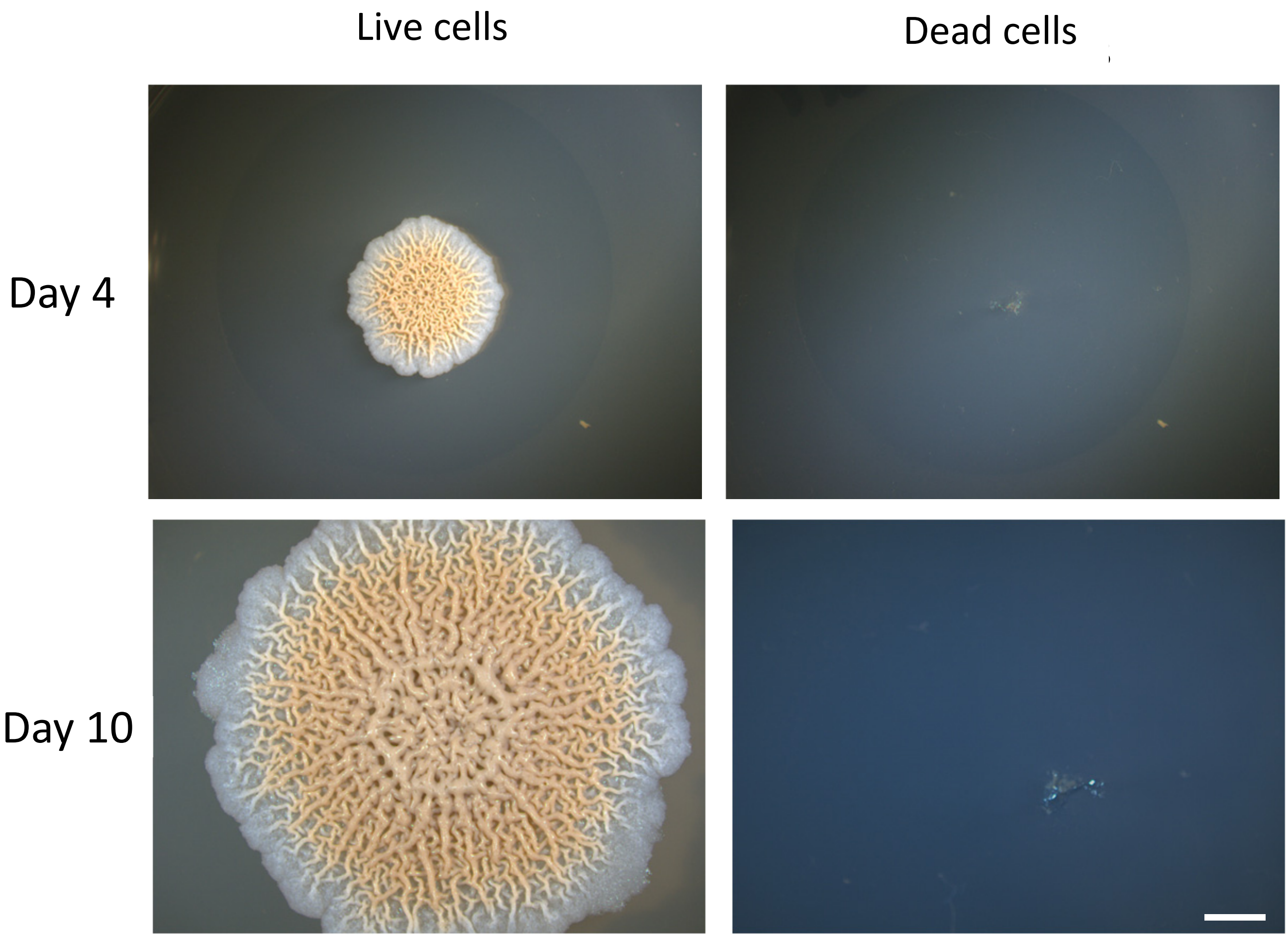

Supplementary Figure S10

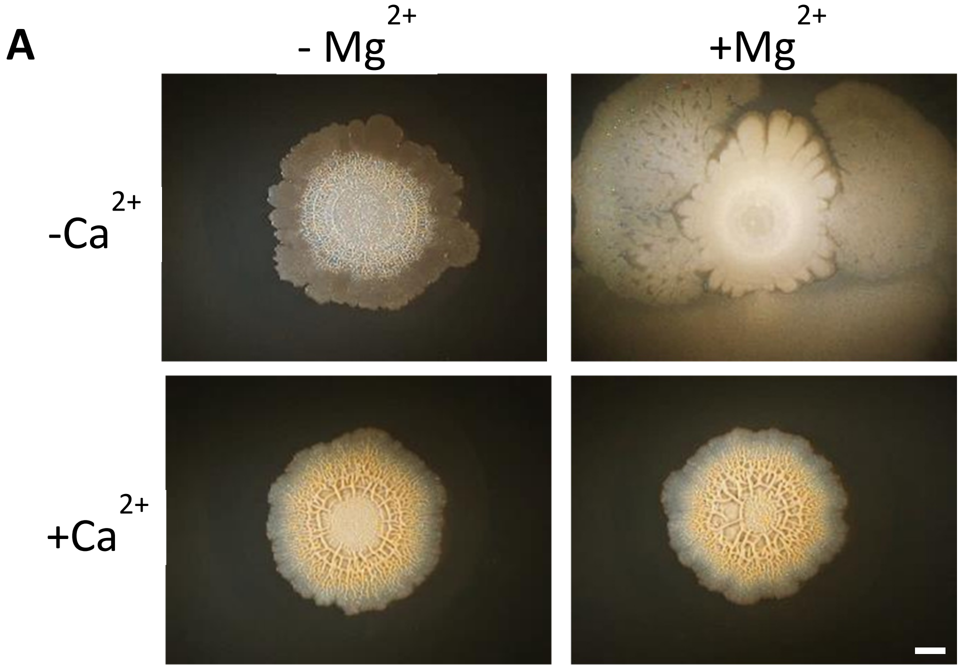

B

mM Ca^{2+}	0	0.097	0.187	0.375	0.75	1.5
0						
1.5 mM Mg^{2+}						
3 Mm Mg^{2+}						
1.5 mM Ba^{2+}						
3 mM Ba^{2+}						

Supplementary Figure S11

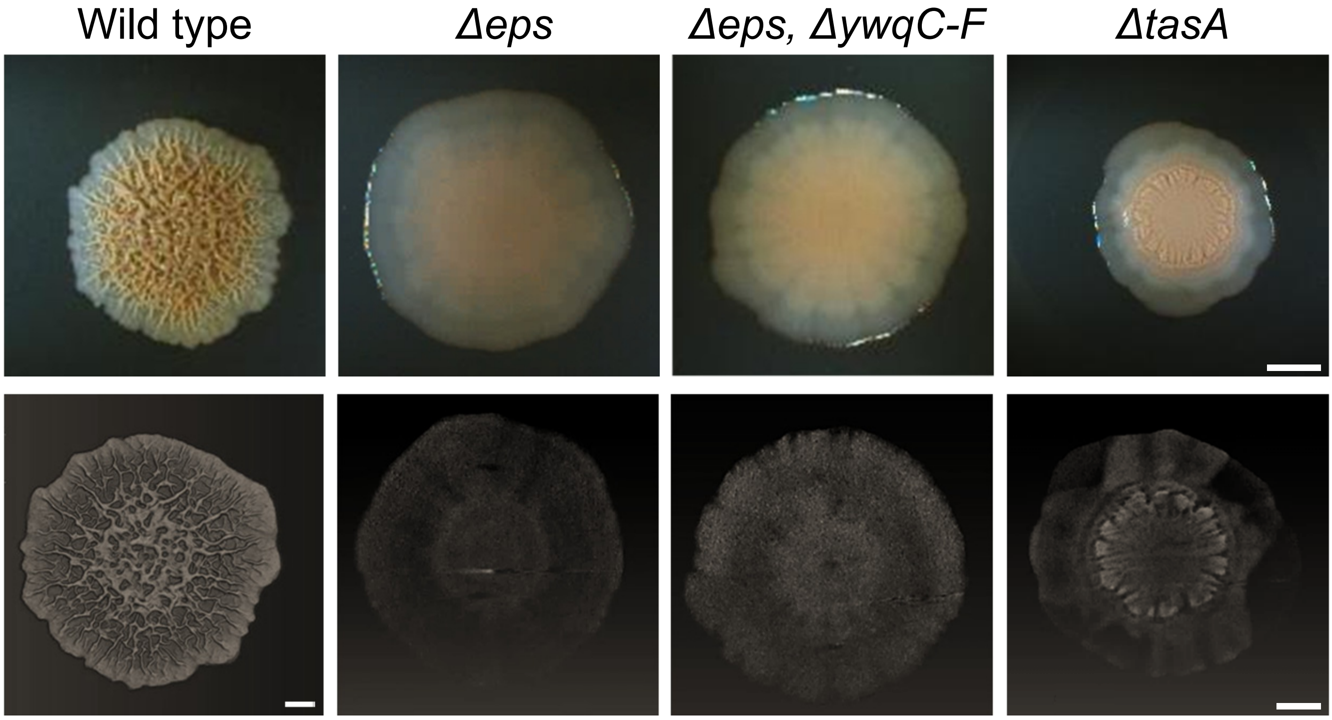

Supplementary Figure S12

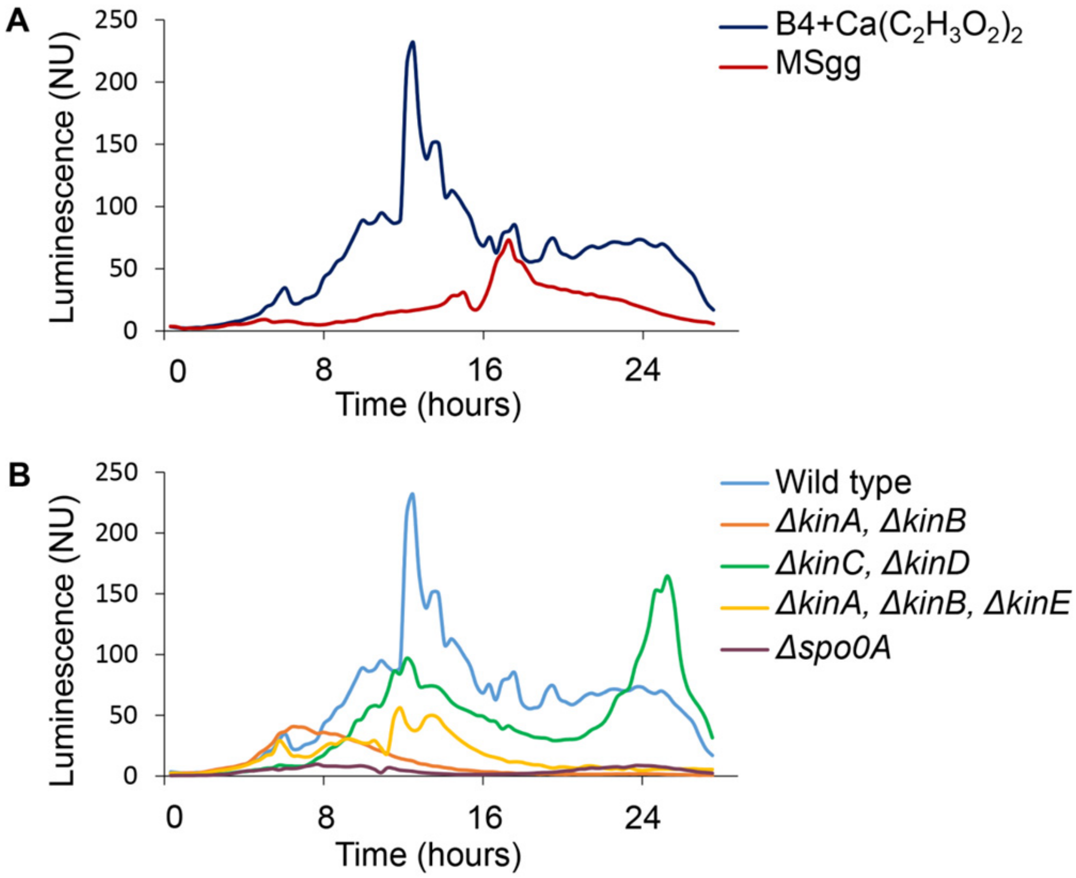

Supplementary Figure S13

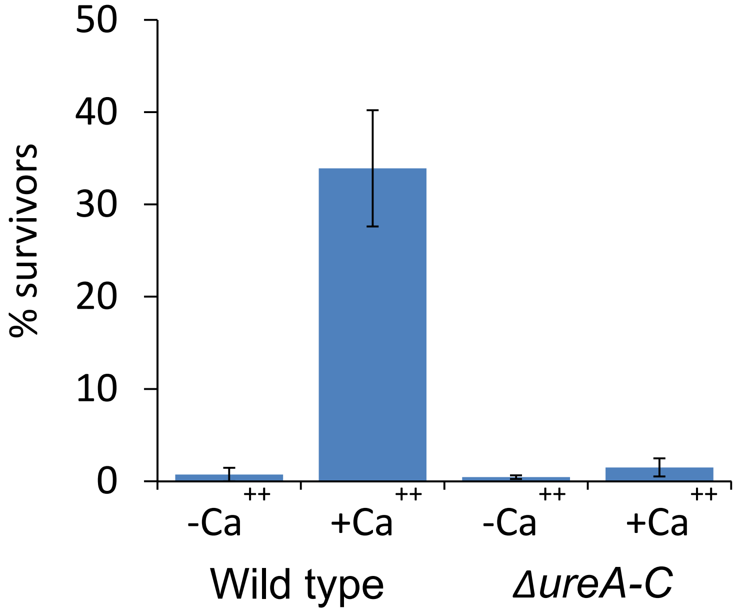

Supplementary Figure S14

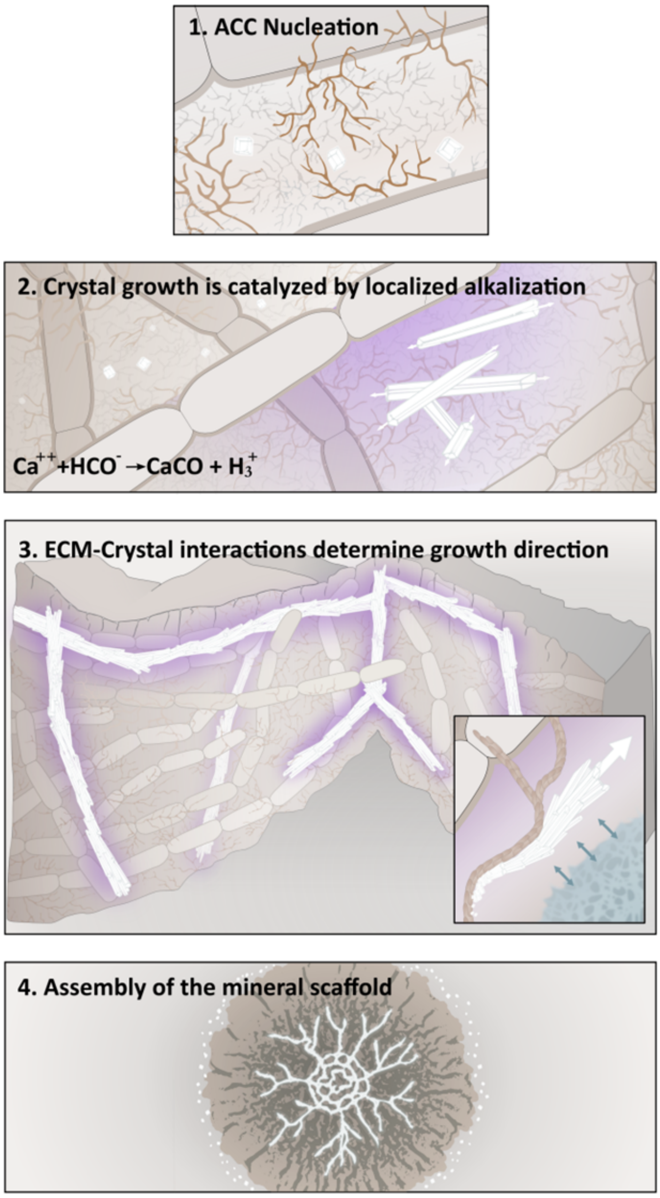

Supplementary Figure Legends

Supplementary Figure S1.

Precipitation of calcite minerals in anaerobic conditions. Top view of biofilms formed by *B. subtilis*. The biofilms were grown on solid biomineralization-promoting medium with (**A-right** and **B**) or without (**A-left**) a calcium source for 7 days, at 30°C. Images show two environmental conditions; anaerobic condition with nitrate source (**A**) and CO_2 enriched environment (**B**). Images were taken by stereo microscope with objective 0.5x. Scale bar corresponds to 2 mm. The results are of a representative experiment out of three independent repeats.

Supplementary Figure S2.

XRD diffraction audiogram of calcite mineral precipitated at the edges of the colony.

The colony was grown on solid biomineralization-promoting medium with calcium source for 21 days at 30°C, in a CO_2-enriched environment.

Supplementary Figure S3.

Sporulation has little or no effect on biomineralization. Top view of the biofilm morphology of a mutant blocked in sporulation (*sigF* null mutant). The biofilms were grown on a solid biomineralization-promoting medium with a calcium source for 5 days at 30°C, in a CO_2-enriched environment. Images were taken by stereo microscope with objective 0.5x. Scale bar corresponds to 2 mm. (**Lower panel**) MicroCT images of the biofilms formed by the *sigF* mutant strain. Scale bar corresponds to 2 mm. The results are of a representative experiment out of three independent repeats.

Supplementary Figure S4.

Mutant for the *lcfA* gene is defective in calcium carbonate precipitation and wrinkles sustainment. (**Upper panel**). Top view of the biofilm morphology of the *lcfA* mutant. The biofilms were grown on solid biomineralization-promoting medium with a calcium source for at 30°C, in a CO_2-enriched environment. Images of biofilm, taken with a Nikon D3. Scale bar corresponds to 1 mm. (**Lower panel**) MicroCT images of the biofilms formed by the *lcfA* mutant strain. Scale bar corresponds to 2 mm. The results are of a representative experiment out of three independent repeats.

Supplementary Figure S5.

Strains defective in biomineralization do not have defects in planktonic cell growth. Growth curves of wild type and its mutant derivatives (*lcfA, epsH*) at 30°C, in liquid biomineralization-promoting medium. Planktonic growth of cells was monitored in a microplate reader by measuring OD_{600}. The results represent the averages and standard deviations of six wells per strain within three independent experiments.

Supplementary Figure S6.

Deletion of Urease has a mild effect on the calcium crystals precipitation. Top view of biofilm morphology of *B. subtilis* wild type and *ureA-C* mutant. Biofilm of the wild type (**Upper panel**) and the *ureA-C* (**Lower panel**) mutant were grown on solid biomineralization-promoting medium with a calcium source and with acidic (pH5.5) and neutral (pH7.0) environment, for 21 days, at 30°C, in a CO_2-enriched environment. Images were taken by stereo microscope with objective 0.5x. Scale bar

corresponds to 2 mm. The results are of a representative experiment out of three independent repeats.

Supplementary Figure S7.

Alignment of putative beta carbonic anhydrase proteins of *Bacillus subtilis* to the beta carbonic anhydrase protein of *Bacillus megaterium*. Shown are the blast alignments of *ytiB* (71% identity) and *yvdA* (64% identity) to a carbonic anhydrase *ytiB* (*Bacillus. megaterium*).

Supplementary Figure S8.

Overexpression of *yvdA* encoding for Carbonic anhydras improves construction formation and noticeable calcite granules precipitation. Top view of biofilm morphology of wild type strain and a *yvdA*-overexpressing strain (*phyper-spank-yvdA-spc*). The biofilms were grown on solid biomineralization-promoting medium with a calcium source, for 7 (**Upper panel**) or 21 (**Lower panel**) days, in 30°C, in a CO_2-enriched environment. Images were taken by stereo microscope with objective 0.5x. Scale bar corresponds to 2 mm. The results are of a representative experiment out of three independent repeats**.**

Supplementary Figure S9.

Dead cell cannot induce biomineralization. Top view of biomineralization plates inoculated with live and dead *B. subtilis* cells. The starter culture was incubation with fumes of hypochlorite in a sub-lytic toxic concentration for 30 min, centrifuged at 4000rpm for 5 min and washed in PBS X2. Then, placed on solid biomineralization-

promoting medium with a calcium source, for 4 (**Upper panel**) or 10 (**Lower panel**) days, in 30°C, in a CO_2-enriched environment. Images were taken by stereo microscope with objective 0.5x. Scale bar corresponds to 2 mm. The results are of a representative experiment out of three independent repeats**.**

Supplementary Figure S10.

Mg^{2+} and Ba^{+2} have little or no effect on wrinkles formation. (A) Top view of the biofilm morphology. The biofilms were grown on solid biomineralization-promoting medium with or without a calcium source and without or without a magnesium source (4mM $MgCl_2$) for 7 days at 30°C, in a CO_2-enriched environment. Images were taken by stereo microscope with objective 0.5x. Scale bar corresponds to 2 mm. **(B) Wrinkles are formed when calcium carbonate is accumulated above a threshold.** Shown is a table of all tested Ca-Actate, $MgCl_2$ and $BaCl_2$ concentrations. The effect of the ions on calcium carbonate precipitation (Yellow), wrinkles morphologies (blue) or both of them (green). The results are of a representative experiment out of three independent repeats.

Supplementary Figure S11.

The extracellular matrix affects amorphous calcium carbonate and calcite distribution (**Upper panel**). Top view of the biofilm morphology of a wild type strain and its derivative biofilm formation mutants (mutants for extracellular matrix production): Single mutants for *eps, tasA* and a double mutant for *eps* and *ywqC-F*. The biofilms were grown on solid biomineralization-promoting medium with a calcium source for 5 days at 30°C, in a CO_2-enriched environment. Images were taken by stereo microscope with objective 0.5x. Scale bar corresponds to 2 mm. (**Lower**

panel) MicroCT images of the biofilms formed by wild type strain and its derivative biofilm formation mutants. Scale bar corresponds to 2 mm. The results are of a representative experiment out of three independent repeats.

Supplementary Figure S12.

The transcription of the matrix activator sinI is synchronized with calcium accumulation. **(A)** Time course of normalized luminescence from a strain harboring *sacA::*P_{sinI}*-luciferase*, grown at 30°C in either biomineralization-promoting medium (B4) with calcium acetate (blue), or in the commonly used biofilm medium (MSgg,(Branda et al., 2001)) (red).

(B) Time courses of light emission of the wild type (azure) and the indicated mutants; *kinA, kinB* (orange), *kinC, kinD* (green), *kinA, kinB, kinE* (yellow) and *spo0A* (purple) harboring *sacA::*P_{sinI}*-luciferase*, grown at 30°C in biomineralization-promoting medium, supplemented with a calcium source. The results are of a representative experiment out of five independent repeats.

Supplementary Figure S13.

Precipitation of calcite minerals has a cardinal role in phenotypic resistance of *Bacillus* biofilms to ethanol. The biofilms were grown on solid biomineralization-promoting medium with or without a calcium source for 3 days, at 30°C in CO_2-enriched environment. Then, biofilms were split into two equal halves and re-suspended either in PBS or in 70% ethanol. After incubation of 20 minutes, the biofilms were centrifuged at 14,000rpm for 5 min and washed in PBS. The number of colony forming units (CFU) was detected by plating the washed samples on LB plates

that were then incubated at room temperature overnight. The percentage of surviving CFUs is represented by the ratio of biofilm cells treated by ethanol/untreated cells.

Supplementary Figure S14.

Model for biomineralization-mediated scaffolding of bacterial biofilms. ACC- amorphous calcium carbonate nucleation (1, see also Figure 2). The mineral growth is facilitated by localized alkylation of the microenvironment (2, see also Figure 3). The directed growth of the mineral scaffolds allows mechanical support of the 3D structure (3). The bacterial amyloids (brown) promote mineral and crystal growth in specific directions, while acidic exopolysaccharides (cyan) antagonize the mineral growth (Inlet within 3. see also Figure 4). Together, the biomineral interactions result in functional structure relying on calcium carbonate scaffolds and mineral deposition at the edges of the colony (4, see also Figure 1, Figure 2 and Figure 6).

Table S1: Strains used for this study

Name in text	Genotype	Reference
Wild type	NCIB3610	Conn H. J. 1930, Branda *et al.* 2001
tasA	*ΔtasA::kan*	Branda *et al.* 2006
eps	*ΔepsH::tet*	Branda *et al.* 2006
ywqC-f	*ΔywqC-F::mls*	lab collections
eps, ywqC-f	*ΔepsH::tet, ΔywqC::mls*	Lab collections
kinA, kinB, p_{sini}*-luciferase*	*ΔkinA::mls,ΔkiBb::kan, sacA::* p_{sini}*-lux (cam)*	Mcloon *et al.* 2011
kinC, kinD, p_{sini}*-luciferase*	*ΔkinC::mls,Δkind::tet, sacA::* p_{sini}*-lux (cam)*	Mcloon *et al.* 2011
kinA, kinB, kinE, p_{sini}*-luciferase*	*ΔkinA::mls,ΔkinB::kan,Δkine::mls,sacA:: sacA::* p_{sini}*-lux (cam)*	Lab collections
lcfA	*ΔlcfA::spec*	This study
ureA-C	*ΔureA-C::mls*	This study
yvdA	*ΔyvdA::mls*	This study
ybcF	*ΔybcF::kan*	This study
ytiB	*ΔytiB::tet*	This study
yvdA, ybcF	*ΔyvdA::mls, ΔybcF::kan*	This study
yvdA, ytiB	*ΔyvdA::mls, ΔytibA::tet*	This study
ybcF, ytib	*ΔybcF::kan, ΔytiB::tet*	This study
yvda, ybcf,ytiB	*ΔyvdA::mls, ΔybcF::kan, ΔytiB::tet*	This study
Overexpression of *yvda*	*amyE::*$p_{hyper\text{-}spank}$*-yvdA-spc*	This study
sigF	*sigF::Kan*	Camp *et al.*, 2011

Table S2: Primers list used for this study

**Name**	**Sequence**
lcfA operon primer A	5'- acg tat cgc ttg aac ttg atc ttc gcg gcg aac -3'
lcfA operon primer B	5'-caa ttc gcc cta tag tga gtc gtt gca taa aac ctc ccc ttt c-3'
lcfA operon primer C	5'-cca gct ttt gtt ccc ttt agt gag gga aat ccc gga ctt taa aag tcc-3'
lcfA operon primer D	5'- ttc tgt ggc tga tcc ttt gcc ctt cc-3'
UreA-C operon primer A	5'- tga ata aat ata aca aaa aaa gaa gct gat ttg gtc aag g -3'
UreA-C operon primer B	5'- caattcgccctatagtgagtcgt ctt ttg tca tat aaa gca gat gcg gct act acg aat ttg c -3'
UreA-C operon primer C	5'- ccagcttttgttccctttagtgag aag caa gtc att aaa aga tgt tat gaa tca tct ctt tta atc -3'
UreA-C operon primer D	5'- caa ata ttc ttt cgg aaa ttc cgg cgt atc cat taa acg g -3'
yvdA primer A	5'- aga ttg ctc cag caa tgt atc aag-3'
yvdA primer B	5'- caa ttc gcc cta tag tga gtc gt ctc cca tcc ata tga ttt tgc aag-3'
yvdA primer C	5'- cca gct ttt gtt ccc ttt agt gag agg gag gtt ata aca aaa tat gcg att c-3'
yvdaA primer D	5'- gaa acc gct ccg ttt ttt ata ttg gtc-3'
**y*bcF*** primer A	5'- ggc atg gca gtc aaa gta cta ata atc-
**y*bcF*** primer B	5'- caa ttc gcc cta tag tga gtc gt gca cac ctc ttc ctt atg ttt atc-3'
**y*bcF*** primer C	5'- cca gct ttt gtt ccc ttt agt gag atg aaa tgc agg ttt aac ttc taa acg c-3'
**y*bcF*** primer D	5'- ctc ata aca aca gcg gat gtt aga tc -3'
**y*tiB*** primer A	5'- cgt acc ttc aaa agc gtg aac att c-3'
**y*tiB*** primer B	5'-caa ttc gcc cta tag tga gtc gt gtt cgt tgt ccc ttt cta ttc ttt tta c-3'
**y*tiB*** primer C	5'- cca gct ttt gtt ccc ttt agt gag agg aac ggg atc gac tcc tc-3'
**y*tiB*** primer D	5'- ttt cat gcc atc acc ctt tca tc-3'
p1-Sali-*yvdA*-fw	5'- aaaa gtcgac aaaggtggtgaactact atg aat caa atg gtt tct tta aca tca att ttg gaa cac-3'
p2-Nhei-*yvdA*-rev	5'- ttt tgc tag ctt atg agt gat tgt tta taa gct cag ttt cat aac c -3'

Supplementary references:

Conn H. J. The identity of *Bacillus subtilis*. J. Infect. Dis. 1930; 46:341–350

Branda, S.S., Chu, F., Kearns, D.B., Losick, R., and Kolter, R. A major protein component of the *Bacillus subtilis* biofilm matrix. *Molecular microbiology.* 2006; 59:1229-1238.

Branda SS, Gonzalez-Pastor JE, Ben-Yehuda S, Losick R, Kolter R. Fruiting body formation by Bacillus subtilis. *Proceedings of the National Academy of Sciences of the United States of America* **98**, 11621-11626 (2001).

McLoon, A.L., Guttenplan, S.B., Kearns, D.B., Kolter, R., and Losick, R.Tracing the domestication of a biofilm-forming bacterium. *Journal of bacteriology.* 2011; 193:2027-2034.

Camp, A. H., Wang, A. F. & Losick, R. A small protein required for the switch from {sigma}F to {sigma}G during sporulation in *Bacillus subtilis*. *Journal of bacteriology* 193, 116-124, doi:10.1128/JB.00949-10 (2011).
